# Alteration of Plasma Amino Acid Concentrations in Iranian Children with COVID-19

**DOI:** 10.1155/2022/9390327

**Published:** 2022-07-29

**Authors:** Sedigheh Shams, Aileen Azari-Yam, Moeinadin Safavi, Zahra Zamani, Maryam Sotoudeh-Anvari, Meisam Sharifzadeh Ekbatani, Mohammad-Taghi Haghi-Ashtiani, Fatemeh Mozafari, Bahareh Yaghmaie, Leila Shafeghat

**Affiliations:** ^1^Pathology Department, Pediatrics Center of Excellence, Children's Medical Center, Tehran University of Medical Sciences, Tehran, Iran; ^2^Growth and Development Research Center, Tehran University of Medical Sciences, Tehran, Iran; ^3^Molecular Pathology and Cytogenetic Division, Pathology Department, Children's Medical Center, Tehran University of Medical Sciences, Tehran, Iran; ^4^Pediatrics Center of Excellence, Children's Medical Center, Tehran University of Medical Sciences, Tehran, Iran; ^5^Division of Pediatric Intensive Care Unit, Pediatrics Center of Excellence, Children's Medical Center, Tehran University of Medical Sciences, Tehran, Iran

## Abstract

COVID-19 is an acute viral disease that has so far infected more than 200 million and killed more than four million worldwide. It affects the immune system and other organs. Here, we investigated the level of free plasma amino acids in COVID-19 patients and compared them with non-COVID-19 subjects. We also compared amino acids levels in critically ill patients admitted to the intensive care unit (ICU) with non-ICU patients and expired and recovered patients. Twenty-six COVID-19 patients and 32 non-COVID-19 subjects were included in the study. The mean of glutamic acid, serine, glycine, threonine, phenylalanine, leucine, lysine, alanine, arginine, aspartic acid, and ornithine was significantly higher in cases than controls. In addition, the mean of glutamine was significantly lower in patients than controls (443.89 ± 254.31 vs. 651.73 ± 107.38, PV < 0.001). Low level of glutamine and isoleucine was seen in the majority of ICU and expired patients, respectively. Logistic regression analysis showed low level of isoleucine as a predictor variable in mortality (*P* = 0.02, EXP (*B*) = 16.5, and CI 95% = (1.48, −183.07)). There was a positive and significant relationship between some amino acids levels, serum liver enzymes, and sodium concentrations. There was also a significant but negative correlation between histidine levels, ESR, and ferritin. Phenylalanine had a highly positive relationship with serum procalcitonin in patients (*R*^2^ = 0.534, PV = 0.015). Our studies have shown the alteration of plasma amino acids concentration in COVID-19 patients. These changes are more evident in critically ill and at-risk patients.

## 1. Introduction

The newly identified coronavirus SARS-COV-2 in 2019, resulting in Coronavirus disease 2019 or COVID-19, has so far infected more than 200 million and killed more than four million worldwide [1]. At the beginning of the pandemic, it was thought that children would not be infected and no deaths were reported. As the prevalence increased, morbidity and mortality were reported in children with lower rates compared to adults [2, 3].

The virus has the potential to damage vital organs including the heart, lungs, liver, and kidneys [4]. The evidence suggests that generalized inflammation and cytokine function are among the leading causes of death. The combination of anti-inflammatory and antiviral drugs seems to have a good effect in inhibiting the secretion of cytokines and preventing its adverse effects on the lungs. [5]

Adequate attention to the nutrition of the hospitalized patient and the use of supplements and modulation of the immune system can reduce inflammatory processes and fatal consequences by reducing infection and cytokine storms. It also shortens hospital stays and reduces costs, which is especially important during a pandemic.

Intake of proteins and amino acid supplements in addition to strengthening the immune system prevents muscle loss and improves respiratory muscles, which is especially important in patients admitted to the ICU, being immobile for a while. Amino acids can also be used as a source of energy by the body which are classified into three groups: essential, nonessential, and conditional amino acids. Conditional amino acids include arginine, cysteine, glutamine, tyrosine, glycine, ornithine, proline, and serine and are usually not essential, except in times of illness and stress. [6, 7] There are few studies on the roles and alteration of amino acids in COVID-19, especially in young children. In this study, plasma free amino acid levels in children with COVID-19 were compared with those in non-COVID children, and its relationship with inflammatory and nutritional factors was investigated. Differences in amino acid concentrations according to the severity of disease also was evaluated.

## 2. Material and Methods

### 2.1. Study Design and Participant

This retrospective and case-control study was designed and carried out at the Children's Medical Center in accordance with the principles of the Declaration of Helsinki and with the approval of the Ethics Committee of the Tehran University of Medical Sciences (authorization number: 2020/39-39).

Twenty-six COVID-19 patients with the age of 16 days to 15 years and 32 non-COVID-19 subjects with the age of 22 days to 16 years from March through June 2020 were included in the study. SARS-CoV-2 infection was confirmed by reverse transcriptase real-time PCR. Non-COVID-19 subjects were selected randomly from the database of the laboratory as controls. Subjects with specific disorders such as PKU (phenylketonuria) and MSUD (maple syrup urine disorder) were excluded.

Clinical data and other laboratory findings of subjects were obtained from the hospital electronic medical records.

### 2.2. Procedure

Plasma amino acids concentrations were determined by reverse-phase high-performance liquid chromatography (RP-HPLC) on a C18 column equipped with a fluorescence detector after precolumn derivatization with o-phthalaldehyde (OPA). Details are explained in the previous article of the author [8]. Briefly 200 *μ*L of plasma was mixed with 50 *μ*L internal standard, and 800 *μ*L methanol and kept at 4° C for 5 minutes. The tubes were centrifuged at 4,000 rpm for 5 minutes. 250 *μ*L of supernatant was mixed with 100 *μ*L of borate buffer followed by 50 *μ*L of OPA/2ME (2-mercaptoethanol) reagent. 50 *μ*L of this solution along with mobile phase and hydrochloric acid injected to the HPLC system (Knauer, Germany). The calibrators and chemicals utilized in the methodology were purchased from RECIPE (chemical+ Instruments GmbH). Internal standard, L-homoserin was purchased from Sigma Aldrich® (St. Louis, USA). Mercaptoethanol and ortho-phthalaldehyde (OPA) and others chemicals and reagents utilized were from Merck® (Darmstadt, Germany). The deionized water used was from the water purification system of TKA smart 2 pure (Germany).

### 2.3. Statistics

IBM SPSS Statistics 21.0 software was used for statistical analysis and *P* value < 0.05 was considered statistically significant. The mean ± SD was used to express the concentration of amino acids. We used independent sample *t*-test with CI95% to compare concentrations of amino acids between two independent groups of patients and cases. Additionally, the concentration of amino acids was compared in two groups ICU and non-ICU patients as well as recovered and expired patients. The Pearson correlation coefficient was used to express the relationship between inflammatory and biochemical factors with amino acid concentrations in patient.

## 3. Results

In the present study, 26 cases and 32 controls were examined and their laboratory findings were compared. The mean age of COVID-19 patients was 6.36 ± 5.07 years (16 d-15 y), and the mean age of controls was 5.55 ± 4.97 years (22 d-16 y). Female : male ratio was 14 : 12 in the patient group, and it was 11 : 21 in the control group. No significant differences between patients and control were seen in regards to age and sex (*P* value = 0.5 and 0.1, respectively). We also classified children in both COVID-19 and non-COVID-19 groups in terms of age: under one year, 1 to 2 years, 2 to 5 years, 5 to 10 years, 10 to 15 years, and above 15 years. Both case and control groups were compared according to the defined age groups, there was no significant difference between them as well as (*P* value = 0.2). None of the controls had an underlying disease, but 3 of the cases (12%) had an underlying disease and the difference was significant (P value < 0.001).

As reported in previous studies at our center [9, 10], the chief complaints of most patients were fever (60%) and cough (40%). Most patients (53%) had positive radiologic findings (CT) in favor of COVID-19, 32% were normal, and 15% had no radiological evaluation. The mean length of hospital stay was 25 days, with maximum of 90 and minimum of 3 days, which differed in ICU and non-ICU patients. Hospitalization duration in ICU patients was 32 ± 27 (7-90) days and in non-ICU patients was 20 ± 20.4 (3-67) days (. Out of 26 COVID -19 patients, seven (26.9%) expired and 19 (73.1%) recovered.

The mean of free plasma amino acids concentrations in cases and controls are presented in Table 1. The mean of glutamic acid, serine, glycine, threonine, phenylalanine, leucine, lysine, alanine, arginine, aspartic acid, and ornithine were significantly higher in cases than controls. In addition, the mean of glutamine was significantly lower in patients than controls. There were no significant differences in asparagine, citrulline, *α*-aminobutyric acid, histidine, isoleucine, methionine, taurine, tryptophan, tyrosine, and valine between patients and controls (*P* value > 0.05).

The frequency of abnormality in plasma concentration of amino acids was calculated using laboratory reference intervals (RI). The results are shown in Table 2. Phenylalanine, glutamic acid, and aspartic acid were higher than the upper limit of RI in most patients (84.6, 76.9, and 76.9%, respectively). In contrast, glutamine and isoleucine were less than the lower limit of RI in about 40% of patients.

Out of 26 COVID-19 cases, 10 (38.5%) patients were admitted to ICU. Plasma amino acids concentration were compared, between ICU and non-ICU patients. The mean of arginine, glycine, isoleucine, *α*-aminobutyric acid, and serine in ICU patients was significantly lower than non-ICU patients (Table 3). Low level of glutamine and isoleucine was seen in 50% and70% of ICU patients, respectively.

We also compared the amino acid concentrations of patients who recovered and those who expired. The results are shown in Table 4. The mean of amino acids alanine, arginine, glycine, isoleucine, and serine was lower in patients who have expired. Low level of glutamine and isoleucine was seen in 57% and 86% of expired cases, respectively. Logistic regression analysis (95% confidence interval) was performed to determine the risk of admission to ICU and mortality prediction, and just isoleucine was considered a predictor variable in mortality (*P* = 0.02, EXP (*B*) = 16.5, and *CI* 95% = (1.48, −183.07)), and its decrease in patients was associated with an increase in mortality.

The correlation of some inflammatory and biochemical factors with plasma amino acids levels in COVID-19 patients were investigated using the Pearson correlation coefficient test. Details are shown in Table 5 (if statistically significant). There was a positive and significant relationship between some amino acids levels, serum liver enzymes, and sodium concentrations.

There was also a significant but negative correlation between histidine levels, ESR, and ferritin. Phenylalanine had a highly positive relationship with procalcitonin.

## 4. Discussion

COVID-19 is an acute viral disease that affects the immune system and other organs, including the liver. The role of nutrition in improving immune system function is well known. Amino acids are involved in various body metabolic pathways as both nutritional and functional factors, and their concentration varies in different health and disease conditions.

In limited studies on SARS-COV-2, the amino acid profiles and the role of some of them in prophylaxis and improvement of the disease have been studied. In this study, we investigated the level of free plasma amino acids in COVID-19 patients and compared them with non-COVID-19 subjects. We also compared amino acids levels in critically ill patients admitted to the ICU with non-ICU patients, expired, and recovered patients.

Of the 22 amino acids that were studied, the mean of 17 was higher in patients than controls. These increases were statistically significant in 11 amino acids. The mean of glutamine was significantly lower in patients than controls. Our results were consistent with some studies and inconsistent with others.

Wu et al. [11] investigated alteration of amino acid profiles in COVID-19 patients during hospitalization and one month after discharge. They found the levels of most amino acids (AAs) including aromatic AAs (tyrosine, phenylalanine, and tryptophan) and branched chains AAs acids (valine, leucine, and isoleucine) significantly were increased in patients compared with controls. The majority of these AAs returned to normal in one month. The level of methionine was significantly lower in patients than controls, and it remained low in patients even after one month. They supposed as liver total protein synthesis capacity is reduced in patients, alteration in AAs originate from the protein breakdown instead of decrease in their clearance. They also concluded that low methionine may be due to high oxidative stress following SARS-COV-2 infection.

Although the concentration of amino acids in our patients was higher than in the control group, it did not mean that they were abnormal in all cases. As shown in Table 2, the frequency of abnormality is different. In our study, the level of *Phe* was higher in patient than controls; meanwhile, it was above the normal range in more than eighty percent of patients. In a study, Huanga et al. [12] showed that phenylalanine and leucine could have prognostic values and identify mortality risk in patients with severe infection. Moreover, it is independent of traditional risk factors. Based on their findings patients with Phe ≥ 84 *μ*M, extremely low level of leucine was at higher risk. The exact mechanisms underlying the elevated phenylalanine in severe infection are unclear. Tissue breakdown, which is probably related to insufficient tissue perfusion, aepsis-induced inflammation, and low tetrahydrobiopterin (BH4), a cofactor of phenylalanine hydroxylases, are some hypotheses.

The mean of *glutamine* was significantly lower in our patients than in controls and was much lower in ICU and expired patients. The amino acids cysteine, arginine, and glutamine are among the amino acids whose role in the immune system has been studied. Glutamine, along with its numerous roles, is known as a substrate for the synthesis of glutathione, a powerful antioxidant.

Cengiz et al. [13] in a study reported the effect of L-glutamine supplementation in shortening the length of hospital stay in the ICU.

There are different opinions about arginine. Although the mean of arginine in our patients was higher than the control group, the level of arginine in ICU and expired patients was lower than in non-ICU and improved patients. Rees et al. [14] in their study showed depletion of arginine in both children with SARS-CoV-2 infection and those with MIS-C. They suggested that arginine depletion might be the cause of coagulopathy, endothelial dysfunction, and T cell dysregulation in COVID-19. Reizine et al. [15] investigated the role of MDSC (myeloid-derived suppressor cells (MDSC) in COVID-19-associated ARDS and its correlation with lymphopenia and increased arginase activity. They suggested that arginine supplementation can play an effective role in improving the length of hospital stay and mortality of ICU patients.

Melano et al. [16], in an in vitro study using human embryonic kidney 293 (HEK293T) cells, investigated the role of lysine, Arg, and their ester derivatives on SARS-CoV-2 and influenza-A virus infection. They studied different steps of virus entry include binding, internalization, endocytosis, and uncoating and observed that these amino acids potentially disturb virus uncoating rather than virus attachment and endosomal acidification. Based on the study findings, they suggested reducing the consumption of arginine-rich foods and lysine supplementation can play a prophylactic role against these viruses. However, their study did not investigate the effects of these two amino acids after virus infection.

Another finding of our study was the low level of Isoleucine as predictor variable for mortality. In 42% of our patients, isoleucine was less than 30uM (RI = 30-130 *u*M), and the mean of ileu was even lower in the ICU-patients and the expired (28 and 21 *μ*M, respectively). The role of branched chain amino acids was investigated in several studies. Muendlein et al. [17] in their study reported low serum valine and leucine levels as mortality predictors in patients with established CVD. Gu et al. [18] in a review article has investigated the role of isoleucine in maintaining the immune system and reduction of rotavirus infection following administration of L-isoleucine in the piglet model. Attenuation of E. coli infection by isoleucine through the modulating antimicrobial peptide expression as well as its effect on plasma level of endotoxin and IL6 in weaned pigs also were reported [19]. However, more studies are needed on the role of branched chain amino acids, especially isoleucine, in the human immune system.

In our study, no strong correlation was found between inflammatory and nutritional factors and amino acid levels, which may be due to the small sample size of patients. Although the negative association between histidine and inflammatory factors was significant, however, we did not find any reason for it in the literature. Suliman et al. [20] reported an inverse correlation of amino acid levels with inflammatory factors in chronic kidney disease (CKD) patients. They supposed systemic inflammatory response independently is the cause of low amino acids concentration in CKD patients. Further studies with larger sample sizes are needed to assess the status of amino acids in COVID-19.

## 5. Conclusion

Metabolism-based assessment is a new approach to identification of the patients who are at high mortality risk. In addition, it can be the basis of medical and nutritional interventions. Amino acids both in terms of energy supply and their role in various metabolic processes and the immune system are important compounds. Their balances in the body can play an effective role in maintaining health and recovery of diseases. Although COVID-19 is a new and unknown disease, strengthening the body and immune system is certainly a basic prerequisite for coping with it. Our studies have shown the alteration of plasma amino acids concentration in COVID-19 patients. These changes are more evident in critically ill and ICU patients. It seems nutritional and pharmacological interventions in this field may reduce the severity of the disease and the duration of recovery.

## Figures and Tables

**Table 1:** Plasma free amino acids concentration in COVID-19 patients (cases) and controls.

Analyte	Cases (*n* = 26)	Controls (*n* = 32)	*P* value	Analyte	Case (*n* = 26)	Controls (*n* = 32)	*P* value
	Mean ± SD (*μ*mol/L)	Mean ± SD (*μ*mol/L)			Mean ± SD (*μ*mol/L)	Mean ± SD (*μ*mol/L)	
Alanine	424.83 ± 174.81	318.05 ± 88.74	0.008^∗^	Isoleucine	37.75 ± 20.84	32.36 ± 11.54	0.24
Arginine	78.34 ± 52.70	50.63 ± 18.27	0.016^∗^	Lysine	188.73 ± 99.49	114.21 ± 26.89	0.001^∗∗^
Asparagine	48.98 ± 24.93	48.20 ± 9.70	0.87	Methionine	62.85 ± 206.55	18.27 ± 5.80	0.22
Aspartic acid	31.41 ± 16.75	4.37 ± 1.40	<0.001^∗∗^	Ornithine	119.09 ± 74.48	59.78 ± 25.5	0.001^∗∗^
Citrulline	25.65 ± 17.65	32.07 ± 12.19	0.11	Phenylalanine	149.72 ± 81.12	60.65 ± 10.58	<0.001^∗∗^
*α*-Aminobutyric acid	18.24 ± 11.11	20.03 ± 10.34	0.53	Serine	198.54 ± 78.31	138.14 ± 25.65	0.001^∗∗^
Glutamic acid	223.1 ± 152.07	58.81 ± 26.40	<0.001^∗∗^	Taurine	71.34 ± 34.22	74.76 ± 25.64	0.66
Glutamine	443.89 ± 254.31	651.73 ± 107.38	<0.001^∗∗^	Threonine	196.34 ± 89.86	124.15 ± 38.88	0.001^∗∗^
Glycine	353.04 ± 126.27	218.23 ± 65.79	<0.001^∗∗^	Tryptophane	54.20 ± 28.27	54.90 ± 12.83	0.90
Histidine	78.78 ± 24.21	68.21 ± 18.11	0.06	Tyrosine	65.6 ± 49.21	55.93 ± 13.84	0.34
Leucine	150.06 ± 72.25	106.91 ± 36.06	0.01^∗^	Valine	232.45 ± 100.69	191.66 ± 51.34	0.069

^∗^ *P* ≤ 0.05 is statistically significant. ^∗∗^*P* ≤ 0.001 is statistically highly significant.

**Table 2:** Frequency (%) of amino acid abnormality in COVID-19 patients according to the Laboratory Reference Interval.

Analyte	Reference interval (*μ*mol/L)	Low *N* (%)	Normal *N* (%)	High *N* (%)	Analyte	Reference interval (*μ*mol/L)	Low *N* (%)	Normal *N* (%)	High *N* (%)
Alanine	240-600	3 (11.5)	20 (76.9)	3 (11.5)	Isoleucine	30-130	11 (42.3)	15 (57.7)	
Arginine	40-160	6 (23.1)	19 (73.1)	1 (3.8)	Lysine	80-250	5 (19.2)	15 (57.7)	6 (23.1)
Asparagine	24-60	4 (15.4)	14 (53.8)	8 (30.8)	Methionine	6-49	1 (3.8)	21 (80.8)	4 (15.4)
Aspartic acid	0-20	—	6 (23.1)	20 (76.9)	Ornithine	20-135		19 (73.1)	7 (26.9)
Citrulline	8-47	1 (3.8)	22 (84.6)	3 (11.5)	Phenylalanine	30-80		4 (15.4)	22 (84.6)
*α*-Aminobutyric acid	6-38	2 (7.7)	22 (84.6)	2 (7.7)	Serine	60-200	1 (3.8)	14 (53.8)	11 (42.3)
Glutamic acid	10-120		6 (23.1)	20 (76.9)	Taurine	19-216		26 (100)	
Glutamine	396-746	10 (38.5)	14 (53.8)	2 (7.7)	Threonine	40-240		20 (76.9)	6 (23.1)
Glycine	140-490	1 (3.8)	22 (84.6)	3 (11.5)	Tryptophan	15-73	1 (3.8)	19 (73.1)	6 (23.1)
Histidine	50-130	3 (11.5)	23 (88.5)		Tyrosine	30-120	3 (11.5)	22 (84.6)	1 (3.8)
Leucine	60-230	1 (3.8)	19 (73.1)	6 (23.1)	Valine	140-350	5 (19.2)	18 (69.2)	3 (11.5)

**Table 3:** Comparison of amino acids concentration (*μ*mol/L) between ICU and non-ICU patients.

Analyte	ICU-patients *N* = 10	Non-ICU patients *N* = 16	*P* value	Analyte	ICU-patients *N* = 10	Non-ICU patients *N* = 16	*P* value
	Mean ± SD	Mean ± SD			Mean ± SD	Mean ± SD	
Alanine	339.54 ± 170.57	478.15 ± 159.97	0.09	Isoleucine	28.01 ± 18.07	43.85 ± 20.62	0.04^∗^
Arginine	54.63 ± 39.15	93.16 ± 55.69	0.04^∗^	Lysine	164.01 ± 74.40	204.19 ± 111.86	0.26
Asparagine	42.98 ± 27.20	52.74 ± 23.51	0.42	Methionine	21.88 ± 20.12	88.46 ± 262.76	0.35
Aspartic acid	26.39 ± 16.10	34.56 ± 16.87	0.26	Ornithine	96.83 ± 34.02	133.01 ± 89.52	0.53
Citrulline	20.60 ± 15.22	28.81 ± 18.78	0.22	Phenylalanine	146.6 ± 83.38	151.67 ± 82.38	0.95
*α*-Aminobutyric acid	13.08 ± 7.80	21.47 ± 11.85	0.05^∗^	Serine	156.61 ± 66.06	224.75 ± 75.49	0.02^∗^
Glutamic acid	242.02 ± 210.15	211.4 ± 107.98	0.91	Taurine	63.65 ± 32.67	75.96 ± 35.38	0.49
Glutamine	344.92 ± 246.23	504.62 ± 246.88	0.11	Threonine	163.07 ± 74.22	217.13 ± 94.63	0.12
Glycine	293.89 ± 135.84	390.01 ± 108.21	0.01^∗^	Tryptophane	44.88 ± 28.39	60.03 ± 27.4	0.14
Histidine	76.94 ± 30.21	79.94 ± 20.63	0.69	Tyrosine	52.70 ± 29.55	73.66 ± 57.72	0.17
Leucine	125.75 ± 56.24	163.74 ± 78.18	0.23	Valine	198.70 ± 87.38	253.55 ± 105.27	0.22

The Mann–Whitney test was used to determine differences between two groups. P ≤0.05 is statistically significant.

**Table 4:** Comparison of amino acids concentration (*μ*mol /L) between the recovered and expired patients.

Analyte	Expired patients *N* = 7	Recovered patients *N* = 19	*P* value	Analyte	Expired patients *N* = 7	Recovered patients *N* = 19	*P* value
	Mean ± SD	Mean ± SD			Mean ± SD	Mean ± SD	
Alanine	287.4 ± 172.7	475.44 ± 149.86	0.03^∗^	Isoleucine	21 ± 8.46	43.93 ± 20.74	0.01^∗^
Arginine	48.62 ± 33.03	89.28 ± 55	0.034^∗^	Lysine	165.25 ± 74.79	197.38 ± 107.65	0.43
Asparagine	32.77 ± 24.56	54.96 ± 22.85	0.08	Methionine	20.04 ± 17.24	78.63 ± 241.20	0.43
Aspartic acid	24.71 ± 18.20	33.88 ± 15.98	0.23	Ornithine	93.17 ± 36.76	128.64 ± 83.04	0.37
Citrulline	17.72 ± 15.38	28.57 ± 17.91	0.12	Phenylalanine	148.20 ± 101.87	150.28 ± 75.36	0.73
*α*-Aminobutyric acid	12.98 ± 9.30	20.18 ± 11.31	0.14	Serine	136.35 ± 52.84	221.45 ± 74.36	0.01^∗^
Glutamic acid	232.02 ± 254.34	219.91 ± 102.53	0.86	Taurine	59.22 ± 28.42	75.80 ± 35.76	0.28
Glutamine	299.98 ± 247.08	495.96 ± 242	0.07	Threonine	174.94 ± 81.06	204.22 ± 93.71	0.47
Glycine	252.17 ± 65.62	390.20 ± 123.67	0.005^∗^	Tryptophane	41.64 ± 25.19	58.83 ± 28.54	0.17
Histidine	78.32 ± 26.86	78.95 ± 23.95	0.84	Tyrosine	46.91 ± 22.16	72.48 ± 54.90	0.24
Leucine	113.48 ± 46.68	161.62 ± 75.95	0.16	Valine	189.87 ± 83.18	248.14 ± 103.97	0.17

The Mann–Whitney test was used to determine differences in amino acids levels between recovered and expired patients. *P* ≤ 0.05 is statistically significant.

**Table 5:** The correlation (*R*^2^) between the concentration of free amino acids and biochemical and inflammatory factors.^∗^PV < 0.05.

Analyte (mean ± SD)	Amino acids (*R*^2^)	Analyte (mean ± SD)	Amino acids (*R*^2^)	Analyte (mean ± SD)	Amino acids (*R*^2^)	Analyte (mean ± SD)	Amino acids (*R*^2^)
Hb (g/dL) 11.29 ± 2.60	His (0.407)	Blood urea nitrogen 18.09 ± 25.09	ASP (-0.413) Phe (0.522)	Magnesium 1.77 ± 0.34	GLU (0.595)	CKMB 18.82 ± 15.64	GLU (-0.715)
ESR (mm) 51.93 ± 45.07	His (-0.585)	Ferritin 2127.64 ± 37.30	His (-0.470)	Procalcitonin 0.54 ± 1.01	Phe (0.534)	Creatinine 0.7 ± 0.46	Phe (0.56) ASP (0.436)
Glucose 116.5 ± 45.31	Orn (0.500) Ala (0.533)	Sodium 135.32 ± 3.61	ASP (0.419) SER (0.526) GLY (0.553) CIT (0.489) TYR (0.430) MET (0.442)	ALT 66.57 ± 135.49	ARG (0.615) VAL (0.423) Isolusin (0.408) ASA (0.418)	AST 57.88 ± 88.68	ASP (0.589) SER (0.527) GLY (0.442) CIT (0.657) TYR (0.790) Tryptophan (0.495) MET (0.956) LYSIN (0.411)

^∗^There were not significant correlation between levels of CRP (44 ± 53.44 mg/L), CPK (83.70 ± 82.14 U/L)), calcium (9.08 ± 0.91 mg/dL), phosphor (4.53 ± 2.64 mg/dL), albumin (3.78 ± 0.69 g/dL), vitamin D (24.19 ± 15.48 ng/mL), uric acid (3.74 ± 1.33 mg/dL), iron (79.12 ± 66.15 *μ*g/dL), ADA (14.55 ± 14.43 U/L), potassium (4.45 ± 0.57 mmol/L), LDH (650.53 ± 325.67 IU/L), zinc (61.47 ± 26.60 *μ*g/dL), ALKP (506.5 ± 620.49 U/L), and any of the amino acids.

## Data Availability

The all data used to support the findings of this study are included within the article.
